# The Power of Information: How Labeling Alters Young Consumer Acceptance and Purchase Intention of Plant-Based Yogurt Alternatives

**DOI:** 10.3390/foods15183350

**Published:** 2026-09-21

**Authors:** Inga Klimczak, Dorota Klensporf-Pawlik, Aleksandra Olejniczak, Sylwia Chudy

**Affiliations:** 1 Institute of Quality Science, Poznań University of Economics and Business, al. Niepodległości 10, 61-875 Poznań, Poland; 2 Department of Dairy Science and Process Engineering, University of Life Sciences in Poznań, ul. Wojska Polskiego 28, 60-637 Poznań, Poland

**Keywords:** plant-based yogurt alternatives, label, consumer acceptance, overall liking, penalty/lift analysis, purchase intention, Check-All-That-Apply, expectation effect, young adults

## Abstract

The growth of the plant-based dairy alternatives market and consumers’ diverse preferences and expectations prompted an evaluation of plant-based yogurt alternatives based on coconut, oat, soy, and cashew nut compared with dairy yogurt. Consumers’ responses were assessed under blind, expected, and informed conditions using hedonic evaluation, the Check-All-That-Apply (CATA) questionnaire, and purchase intention measurement. The type of raw material significantly influenced the physicochemical properties, sensory profile, and consumer perception of fermented products. Dairy yogurt achieved the highest overall liking, while the coconut-based yogurt alternative was the most preferred plant-based product and showed sensory characteristics closest to dairy yogurt. Taste was identified as the main driver of overall liking, followed by creaminess, thickness, and light color. Conversely, artificial flavor notes, blandness, gray appearance, and thin texture were the main negative drivers of overall liking. The integration of physicochemical, sensory, and hedonic data confirmed that consumer responses are shaped through sensory perception rather than direct evaluation of instrumental quality parameters. Product information positively influenced consumer expectations, but its effect on final acceptance depended on whether sensory quality met those expectations. Higher overall liking consistently predicted higher purchase intention, indicating that sensory acceptance remained the primary determinant of consumers’ willingness to buy plant-based yogurt alternatives.

## 1. Introduction

The global plant-based dairy alternatives market was valued at around USD 36.8 billion in 2025 and is expected to reach almost USD 96 billion by 2033. Plant-based beverages dominate the category, but it is yogurt alternatives that make up the fastest-growing subcategory [1]. Retail data from mature European markets such as Germany and France indicate relatively limited growth for plant-based yogurt alternatives, whereas stronger growth has been observed in Spain, the Netherlands, and the United Kingdom, reflecting differences in the stage of market development across European countries [2]. European consumers who reduce their meat consumption tend to also consume plant-based yogurt alternatives more frequently. In Europe in 2023, plant-based yogurt alternatives were the second most frequently consumed plant-based food among flexitarians, next to plant-based beverages [3]. The shift toward yogurt alternatives may be due to their potential to reduce the negative environmental impacts associated with dairy cattle farming, which is recognized as resource-intensive, especially significant land requirements, but also water and feed inputs contributing to greenhouse gas emissions [4,5]. This growing interest is also driven by health consideration, since plant-based yogurts are an alternative source of protein for vegan and lactose-intolerant consumers [6]. A variety of different plants can be used to develop fermented plant-based dairy analogs including legumes, seeds (mainly oilseeds), nuts, grains (cereals and pseudo-cereals), and even fruit pulps [7,8]. These ingredients affect not only the nutritional value of a plant-based yogurt alternatives but also the texture and flavor. Comparing to dairy yogurt, plant-based alternatives contain unique phytochemical components [9] and dietary fiber [10], but most of them have a low protein content, except for chickpea and soy [5,11]. Moreover, undesirable flavors like beany, nutty, and painty, identified previously in plant-based yogurt alternatives, may lower the consumer acceptance, just like watery, thin, and separated texture [12,13,14,15]. Textural properties, especially gel structures and water retention, are weaker in plant-based yogurt alternatives; therefore, the addition of stabilizers is more common than in dairy products [16]. Flavor is recognized as an important limiting factor of acceptability and marketability in plant-based products [17]. Currently, the sensory quality of plant-based plain yogurt is rather poor, and improving it remains crucial. Jaeger et al. [18] reported that even the common strategy of providing information about environmental and health benefits is not enough to overcome the low sensory acceptability of plant-based yogurt alternatives. Lactic acid bacteria fermentation seems to be an eco-friendly, safe, and gentle solution for off-flavor degradation and the production of volatile compounds, which give the final product a richly layered flavor profile [19]. In fermented plant-based products, it also improves functional, nutritional, and textural properties [8].

If the sensory quality of food does not meet consumer expectations, it could result in reduced overall acceptance [20]. Sensory characteristics of food are recognized as key factors in consumer choice and overall liking. Expectations play an extremely important role in food acceptance; they are mainly based on previous experience with a similar product, especially visual appearance and information from the product’s packaging and label [21]. The effects of packaging, branding, labeling, and judgements have been attributed to sophisticated psychological mechanisms, mainly cognitive expectations, which influence actual product judgements. Consumers’ evaluation of food products according only to their preferences and sensory attributes may change significantly when information about the product is disclosed. This strong effect of information on liking after tasting is known in the literature as a disconfirmation phenomenon. The perception of a sensory attribute can be affected both positively and negatively [22]. Using extrinsic variables to improve the receptiveness of a food product is one of the strategies that influences consumer choice, acceptance, and consumption through the process of “sensation transfer”, in which extrinsic attributes are transferred to our sensory perception of a product [23]. Probably, never before has so much information been provided to consumers, and they may feel overloaded with it. Berger et al. [24] demonstrated that more information may play an extremely important role in improving the quality of consumers’ decisions. Large amounts of information can aggravate the decision difficulty, therefore resulting in decision delay and more inefficiency [25,26]. Lee and Lee [27] reported that even as the amount of information increases, consumers might feel more negative emotion and less decision satisfaction. When the information level is low, consumers rely more on the price or brand. The way plant-based products are described can significantly shape perceptions of quality and hedonic responses; plant–dairy blend yogurts were evaluated by consumers differently depending on whether products were labeled as “plant-based” or “dairy” [28]. However, blind sensory evaluation alone is not sufficient to predict consumers’ responses to commercially available products, particularly when the expectations generated by product information interact with subsequent sensory experiences. The consequences of this interaction for both acceptance and purchase intention of plant-based yogurt alternatives remain less well understood.

Therefore, the main research questions of the study were as follows:

(i). How do consumers evaluate the overall liking and sensory quality attributes of yogurt and plant-based yogurt alternatives under blind tasting conditions?
(ii). Which sensory attributes characterize yogurt and plant-based yogurt alternatives, and which of them act as (positive or negative) drivers of overall liking under blind conditions?
(iii). How do ingredients and nutritional information influence expected and informed overall liking of yogurt and plant-based yogurt alternatives?
(iv). Do expectations generated by ingredients and nutritional information lead to disconfirmation, assimilation, or contrast effects in overall liking?
(v). How does purchase intention change between expected and informed conditions, and to what extent is it predicted by overall liking?

## 2. Materials and Methods

### 2.1. Samples

Commercial dairy and plant-based yogurt alternatives from European Union producers were selected for the study. Dairy yogurt and plant-based yogurt alternatives with different plant bases (cashew, coconut, oat, and soy) were purchased based on their regional availability. All fermented products were plain to eliminate potential variability in sensory characteristics due to different flavors and add-ins. The commercial names and brands are not shown. Samples were kept at 4 °C until testing. A detailed description, including ingredients and nutritional composition of the samples, can be found in Table 1. Samples were labeled according to European law (EU Regulation 1169/2011) [29]; all information is accessible to all market participants and does not disclose the company’s know-how. All measurements were carried out in triplicate.

### 2.2. Acidity Determination

The active acidity of fermented products was measured using the pH meter CP-411 (ELMETRON, Zabrze, Poland), which was calibrated before the measurement. Measurements were carried out at room temperature, and the results were expressed in pH units.

Titratable acidity was determined based on the neutralization of acids present in a sample with 0.1 mol/L NaOH, following the guidelines of the Polish standard [30]. Based on the result, the lactic acid percentage (LA%) was calculated.

### 2.3. Color

The optical properties of fermented products were quantified using the compact spectrophotometer Konica Minolta CM-600d (KONICA MINOLTA Inc., Osaka,, Japan) with a D65 xenon lamp as a light source and 10° angle observer. The results of colorimetry can be defined as CIELab (L*, a*, and b*) color indices. The L* index is the lightness value of the sample on the 0–100 scale, with 0 being black and 100 being white. The a* index expresses the position in the color space of a color on the green-red axis and b* on the blue-yellow axis. Negative a* is green, and positive a* is red; negative b* is blue, and positive b* is yellow. Before starting the measurements, the instrument was calibrated on a white standard. The color difference among samples (ΔE* _ab_) was calculated according to the CIE76 formula (Equation (1)).

(1) $${\Delta E}_{ab}^{*}=\sqrt{\left\{{\left(\Delta L^{*}\right)}^{2}+{\left(\Delta a^{*}\right)}^{2}+{\left(\Delta b^{*}\right)}^{2}\right\}}$$

and the Hunter Whiteness Index (1960) [31] was calculated as (Equation (2)):

(2) WI = L* − 3b*

### 2.4. Syneresis

Syneresis analysis of fermented products was performed by centrifugation according to the method of Żbikowska et al. [32]. Briefly, 5.0 g of sample was centrifuged at 2500 rpm for 20 min at 7 °C. The supernatant was weighed, and the syneresis index was calculated according to Equation (3):

(3) Syneresis (%) = (supernatant weight (g)/total weight (g)) × 100%

### 2.5. Sensory Consumer Evaluation

The sensory experiment with consumers took place in a sensory testing laboratory in 2024 at the Poznań University of Economics and Business (PUEB), in accordance with ISO 8589:2007 [33] requirements and with other relevant institutional guidelines and regulations. A total of 77 untrained consumers belonging mainly to young adults’ generation (93.5%), 63.5% of females and 36.5% of males, took part in this study (Table 2).

Participants were recruited using peer-to-peer contacts and social media. They were asked about food allergies and lactose intolerance to qualify them into the study. The experiment ensured anonymity. Before participating in the study, all participants provided informed consent (confirming the terms and conditions) and were informed that participation was voluntary and anonymous and that they could withdraw from the study at any time without providing a reason. No sensitive personal data were collected, and the data were de-identified and analyzed only in aggregate form. After the completion of each session, participants were instructed to refrain from discussing the details of the study after leaving the testing room. All participants were given a small gift for their participation in the study.

### 2.6. Design and Procedure

In the first part of the experiment, all participants were asked to answer questions about their sociodemographic characteristics and whether they have any type of food allergy or lactose intolerance. They were informed about the product, instructed on the testing procedure, and asked to fill out paper forms. All participants took part in 3 separate test sessions (blind, expected, and informed). Brand names and specific information about the products were unknown to the consumers at the first stage of the experiment to minimize potential bias and allow for a more authentic assessment of the intrinsic quality of all products. In the second part of the experiment, participants saw the products’ nutritional information and ingredients (Table 1), and at the third stage, they had full information about the products tested. Participants tasted the samples only during the first and third sessions.

Participants first assessed the liking of the quality attributes on a 9-point hedonic scale, where 1 indicated “extreme dislike” and 9 indicated “extreme like”, and then determined the sensory characteristics of fermented products using the Check-All-That-Apply (CATA) questionnaire. In a familiarization session, participants received comprehensive instructions about the CATA task and the testing process. The questionnaire consisted of 18 terms, each related to different sensory attributes. These attributes were derived from an extensive literature review [28,34] and were further refined by a panel of trained and experienced experts from the PUEB. The attributes included in the CATA were randomized to ensure the validity of our questionnaire and mitigate any potential bias that could arise from the presentation order [35].

A 5-point Likert scale was used to assess purchase intention. where 1 meant “definitely not” and 5 meant “definitely yes.”

The samples were served at a temperature of 15 °C, a total of 20 g (about 2 tablespoons), ensuring that consumers could re-taste the sample several times during session. Odor-free and clear plastic cups were used in the study. Samples were presented in monadic sequence, coded with random three-digit numbers, following a balanced randomization order to avoid the influence of sample ordering [36]. Samples were gently stirred to overcome any liquid separation that may have occurred before sample presentation.

During all sensory evaluation sessions, consumers were offered either still or sparkling water to cleanse their palates, along with dry, unsalted breadsticks to minimize carry-over effects from one sample to another.

### 2.7. Data Analysis

Statistical analyses were performed using XLSTAT 2023.1.6 (Addinsoft, Paris, France) and Statistica 13.3 (TIBCO Software Inc., Tulsa, OK, USA). The significance level was set at α = 0.05.

Physicochemical data were analyzed using one-way analysis of variance (ANOVA). Tukey’s HSD test was used for post hoc comparisons between product means. Hedonic liking data were analyzed using repeated-measures ANOVA (RM-ANOVA). Where appropriate, pairwise comparisons were adjusted using the Holm procedure, and generalized eta-squared (ηG^2^) was reported as the effect-size measure. Relationships between overall liking and individual sensory quality attributes under blind conditions were examined using Pearson’s correlation coefficients.

CATA data were analyzed using Cochran’s Q test followed by McNemar pairwise comparisons. The overall association between products and descriptors was assessed using the chi-square test, and correspondence analysis based on chi-square distances was used to visualize their relationships. Pearson correlations and penalty/lift analysis were used to identify sensory descriptors associated with increased or decreased overall liking.

Multiple Factor Analysis (MFA) was used to integrate three active data blocks comprising sensory liking, CATA descriptors, and physicochemical characteristics. Prior to the global analysis, each block was analyzed separately and normalized according to its first singular value to balance the influence of the different data blocks. The normalized blocks were subsequently combined in the global MFA. Overall liking under blind conditions was included as a supplementary variable and therefore did not contribute to the construction of the MFA dimensions. Similarity between the configurations provided by the active data blocks was quantified using RV coefficients, ranging from 0 to 1, with higher values indicating greater similarity between the multivariate configurations.

Expectation effects were evaluated using expected-minus-blind, informed-minus-blind, and informed-minus-expected differences. Individual assimilation and contrast responses were classified using the direction of the (I − B)/(E − B) ratio; zero or undefined ratios were classified as no effect or unclear responses.

Purchase intention was analyzed using Friedman’s test to compare products and Wilcoxon signed-rank tests to compare expected and informed conditions. Relationships between overall liking and purchase intention were examined using Spearman’s rank correlations separately for each product under expected and informed conditions.

Linear mixed-effects models were used to examine whether overall liking predicted purchase intention. Separate models were fitted for the expected and informed conditions, with purchase intention as the dependent variable and overall liking and product as fixed effects. An additional model examined whether the informed-minus-expected change in overall liking predicted the corresponding change in purchase intention. The consumer was included as a random intercept to account for the non-independence of the five product evaluations provided by each participant. Models were estimated using restricted maximum likelihood (REML), and degrees of freedom were estimated using Satterthwaite’s approximation. Model assumptions were evaluated by examining residual distributions and residual-versus-fitted plots.

## 3. Results and Discussion

### 3.1. Physicochemical Characteristics of Dairy and Plant-Based Yogurt Alternatives

In the present study, selected physicochemical characteristics—pH, titratable acidity, color, and syneresis—were determined. The results obtained indicate that the type of raw material used was one of the main factors determining the physicochemical properties and visual characteristics of the fermented products analyzed (Table 3).

The pH is a crucial quality parameter of yogurt, assessing the acid development of conventional dairy products, and is recognized as the fermentation endpoint [37]. The pH values ranged from 3.97 to 4.35 for cashew and soy products, respectively. There were no significant differences between dairy and oat-based yogurt alternatives, which aligns with findings reported by Oldano et al. [38], who observed that oat-based yogurt alternatives had a similar pH to dairy Greek yogurt. However, similarly to Daszkiewicz et al. [39], fermented coconut-based products had higher pH values compared to dairy yogurt. A slightly different trend was observed in research conducted by Trajkovska et al. [40], where the highest pH values were obtained for almond-based yogurt alternatives, whereas the lowest were for oat. In coconut, soy, and cashew, the pH was observed at levels of 4.66, 4.63, and 4.61, respectively. Titratable acidity expressed as the lactic acid percentage ranged from 0.11 to 1.29% in oat-based and dairy yogurt, respectively. The lactic acid content of plant-based yogurt was significantly lower than in dairy yogurt. The absence of lactose in non-dairy yogurt, which is the most effective substrate for lactic acid bacteria, is the main reason for the lower LA content. Lactic acid not only influences yogurt’s characteristic acidic taste but also affects syneresis and deterioration of product consistency [41]. The differences in titratable acidity among fermented dairy and plant-based products may also reflect the specific product composition (Table 1). Different proteins, organic acids, minerals, sugar types, bacteria, and fermentation conditions have an impact on the buffering capacity and acidic metabolites [42,43].

Color is one of the factors determining consumers’ acceptance of a product. Color analysis revealed that dairy yogurt exhibited the highest brightness (L* = 74.98) value and the most negative a* value (−2.98), associated with the presence of green color, compared to plant-based alternatives. These results are in agreement with the results reported by Grasso et al. [12]. Yogurt brightness is strongly connected to the capacity of proteins and fat globules to scatter and reflect light [44]. According to Cadwallader [45], brightness is a critical quality attribute for dairy products that strongly influences consumer perception and acceptance. Among fermented plant-based products, soy- and cashew-based yogurts had the highest brightness. The opposite was oat-based yogurt alternatives, with the lowest brightness (L* = 61.85) value, less negative a* value (−0.69), and highest positive b* value (10.56), associated with yellow color. The a* values were negative for all samples, indicating a shift in hue toward green, whereas the b* parameter, reflecting the proportion of yellow hue, was positive for all tested products. Further statistical analyses revealed that there were significant differences between fermented samples, except for the a* values for coconut and oat. The highest calculated Hunter’s Whiteness Index (55.05) was observed for dairy yogurt, followed by coconut, cashew, soy, and oat. The total color difference ΔE calculated for plant-based products relative to dairy yogurt revealed that oat-based (13.90) and coconut-based yogurt alternatives (13.16) are most different in their color from the dairy product. The smallest difference was observed for the soy-based yogurt alternative (5.35). It means that for cashew and soy, noticeable differences are observed, and the color shift is easily recognized, whereas for oat and coconut, colors appear as completely different hues. Our results confirmed the previous work of Marlapati et al. [16], where a similar tendency for color parameters among different fermented products was observed. The whiteness index (WI) reached the highest value in the dairy product (55.05), confirming its brightness and whiteness. Lower values were observed for coconut > cashew > soy > oat-based yogurt alternatives. In contrast, Oldano et al. [38] reported that in terms of whiteness, fermented coconut-based products were closer to dairy, followed by oat and soy-based yogurt alternatives. All WI values determined in fermented products were lower than 100, which indicates deviation towards yellowish white. There were no significant statistical differences between cashew and soy.

The instability of water, recognized as the syneresis percentage, is diverse according to the product base. It is undesirable in yogurt and occurs mainly during storage. The highest syneresis was observed for the cashew-based yogurt alternative (27.6%), while the dairy product showed a slightly lower value (25.8%). A complete absence of syneresis was observed in the oat-based product, indicating the highest structural stability. It can be attributed to oats’ high β-glucan content, which increases its water-holding capacity and the stability of the fermentation gel structure [46]. Unlike milk proteins, which form a gel that shrinks when acidified, the soluble fiber components in oats act as natural hydrogels. Trajkovska et al. [40] showed that coconut-, soy-, almond-, and oat-based alternatives have exceptional water-holding capacity, close to 100%, excluding oat-based yogurt alternatives. However, plant-based yogurt alternatives often require the addition of modified starches or other additives like thickeners or stabilizers to enhance the water-holding capacity and to imitate the texture and structural stability of fermented dairy products [39].

### 3.2. Consumer Acceptance Under Blind Conditions

Under blind conditions, young consumers evaluated the products solely based on their sensory properties, without any information about ingredients or nutritional value. This approach enabled the assessment of the intrinsic sensory appeal of dairy yogurt and plant-based yogurt alternatives.

Young consumers significantly differentiated the products in terms of overall liking (F(4.304) = 36.74, *p* < 0.05, and ηG^2^ = 0.237). Dairy yogurt received the highest overall liking score (6.39), significantly exceeding all plant-based alternatives (Table 4). Among the alternatives, young adults rated the coconut- and oat-based products (scores of 5.10 and 4.91, respectively) significantly higher than other yogurt alternatives.

Statistical analysis revealed the significant effect of the product on the degree of acceptance of all organoleptic attributes (*p* < 0.05): color (F(4.304) = 130.63; ηG^2^ = 0.543), thickness (F(4.304) = 47.19; ηG^2^ = 0.305), smoothness (F(4.304) = 42.05; ηG^2^ = 0.272), aroma (F(4.304) = 22.58; ηG^2^ = 0.187), and taste (F(4.304) = 50.82; ηG^2^ = 0.327). Attribute liking scores are presented in Table 4. Young consumers rated the color of the coconut-based alternative and the dairy yogurt as the most desirable, with mean scores of 7.60 and 7.34, respectively, and did not differentiate significantly between them. The oat-based yogurt alternative received the lowest color-liking score (3.01), differing significantly from all other products. Young consumers also rated the coconut-based alternative and dairy yogurt the highest in terms of desired texture-related attributes. In contrast, the thickness and smoothness of the oat- and soy-based yogurt alternatives were rated the lowest. Dairy yogurt and the coconut-based yogurt alternative also received the highest aroma ratings (mean scores of 6.00 and 5.97, respectively), whereas the cashew-based yogurt alternative received the lowest. Pronounced differences were also observed in taste liking. Dairy yogurt achieved the highest taste score (5.96). Among plant-based products, the taste of coconut- and oat-based products was the most acceptable to young consumers, scoring 4.78 and 4.81, respectively. The cashew-based yogurt alternative received the lowest score (2.60). Studies by other authors have also shown that dairy yogurt is generally more acceptable to young consumers than plant-based yogurt alternatives [12,13,47]. Similar to the present study, Grasso et al. [12] reported high young consumer acceptance of a coconut-based yogurt alternative, which was the only fermented plant-based product rated comparably to dairy yogurt. In contrast, the unflavored soy- and cashew-based alternatives received lower acceptability scores. However, these findings differ from those of Gupta et al. [48], who reported that a soy-based yogurt alternative was rated similarly to the reference dairy yogurt in terms of appearance, taste, and overall liking, while the coconut-based product received lower young consumer ratings. Pearson correlation analysis (n = 385) revealed significant positive associations between overall liking and all sensory attributes (*p* < 0.001). Taste showed the strongest association with overall liking (r = 0.750). Weaker, though still significant, correlations were found for thickness (r = 0.372), aroma (r = 0.362), smoothness (r = 0.340), and color (r = 0.338). These results indicate that although visual and textural properties contributed to consumers’ evaluations, taste played the dominant role in shaping consumers’ overall liking under blind conditions.

### 3.3. Sensory Drivers of Consumer Acceptance

The CATA method was used to determine how young consumers perceived the sensory profiles of dairy yogurt and plant-based yogurt alternatives under blind conditions. Young adult consumers typically selected 4 to 5 descriptors per sample, with “light color”, “thick”, “gray color”, “sour”, “bland”, “creamy”, “thin”, and “sweet” being the most frequently chosen (Table 5).

**Figure 1:**
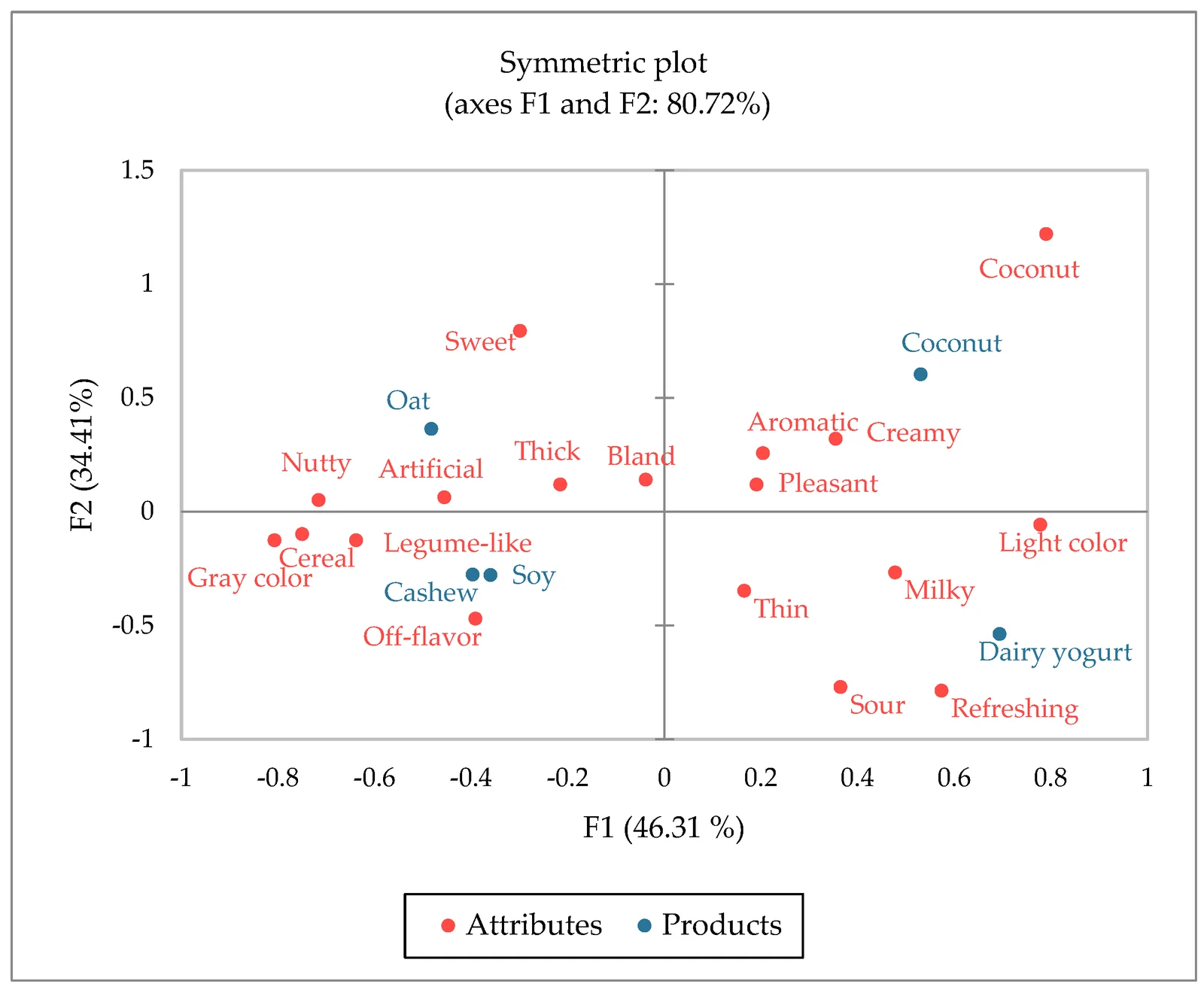
Correspondence analysis (CA) symmetric plot showing the relationship between the evaluated products and the sensory attributes used by consumers.

A significant association was found between products and the CATA attributes (χ^2^ = 985.24, df = 68, and *p* < 0.0001), and Cochran’s Q test confirmed significant differences among products for every attribute (*p* ≤ 0.001), showing that young consumers clearly distinguished the sensory profiles of dairy yogurt and the plant-based yogurt alternatives. Although “sour” was one of the attributes that most strongly differentiated the products (Figure 1), it was not significantly associated with overall liking (penalty/lift, *p* = 0.063). This finding suggests that young consumers perceived sourness as a characteristic yogurt attribute rather than as a determinant of preference.

The correspondence analysis showed that the first two dimensions explained 80.72% of the total inertia, with F1 and F2 accounting for 46.31% and 34.41%, respectively (Figure 1).

Dairy yogurt was mainly associated with “sour”, “milky”, “refreshing”, and “light color”, while the coconut-based alternative was linked to “coconut”, “creamy”, “aromatic”, and “pleasant”, distinguishing its sensory profile from those of the other plant-based alternatives. The oat-based alternative was characterized mainly by “sweet” and “thick” and was located near the descriptor “artificial”, reflecting a sensory profile distinct from both conventional yogurt and the other alternatives. The soy- and cashew-based alternatives were associated with “off-flavor”, “legume-like”, “cereal,” and “gray color”, forming a sensory profile clearly separated from that of dairy yogurt.

Jaeger et al. [34], using CATA, also observed clear differences among plant-based yogurt alternatives. Coconut-based products were characterized by white or off-white color, although their texture varied considerably, from smooth to lumpy and grainy. The soy-based sample was distinguished by a thin and runny appearance, a smooth/creamy yet sticky mouthfeel, and sweet and fruity flavors. One of the cashew-based samples was associated with a light brown or tan color and pronounced sour or acidic notes, whereas products containing an oat–rice–coconut blend were generally thicker and pale yellow. However, direct comparison with the present study is limited because the samples evaluated by Jaeger et al. [34] represented vanilla-flavored variants or were prepared by blending commercial products. These results partly agree with the present findings, confirming that plant-based yogurt alternatives based on different raw materials have distinct sensory profiles. However, the soy-based product evaluated by Jaeger et al. was more strongly associated with sweet and fruity flavors than the soy-based alternative in the present study, which was characterized primarily as legume-like. Similarly, one of their cashew-based samples was distinguished mainly by brown color and acidity. These differences may reflect the use of vanilla flavoring and differences in product formulation, including the use of blended products. Contrasting results were reported by Gupta et al. [48], who also used CATA and integrated the sensory data with compositional and textural measurements using MFA. Their plain soy-based yogurt alternative was positioned close to the dairy reference and on the side of the MFA space associated with positive texture-related descriptors, including “good texture”, “smooth”, and “creamy”. In contrast, the plain coconut-based yogurt alternative was located away from the dairy reference and on the side associated with the descriptors “light” and “artificial”. This pattern differs from the present study, where the coconut-based yogurt alternative was associated with “creamy”, “aromatic”, and “pleasant”, whereas the soy-based product was positioned closer to “off-flavor”, “legume-like”, and “gray color”. These contrasting profiles indicate that products based on the same plant source may differ substantially depending on their commercial formulation, composition, and textural structure.

**Figure 2:**
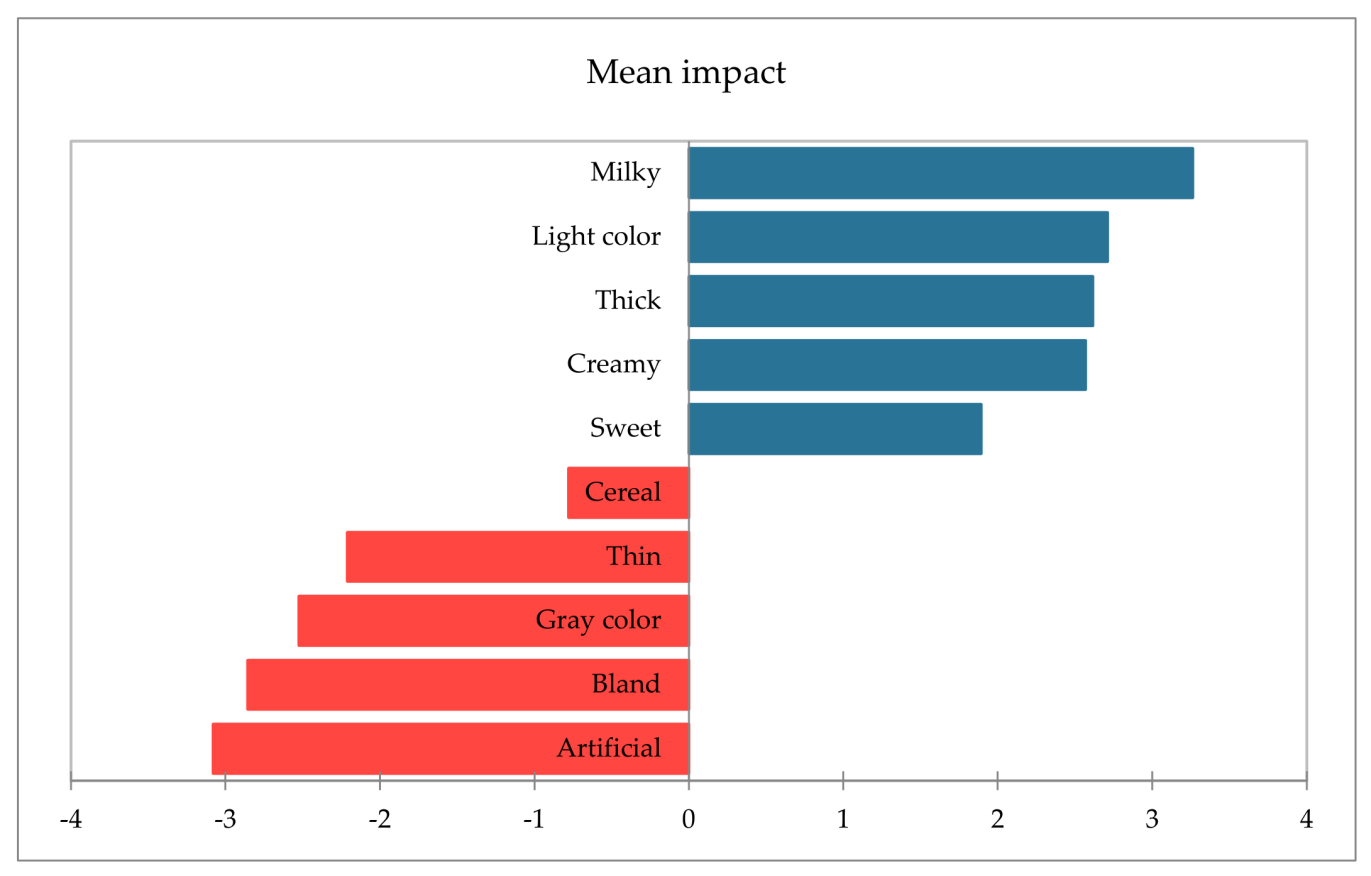
Penalty/lift analysis: mean impact of CATA attributes on consumers’ overall liking under blind conditions. Values represent the difference in mean overall liking (blind condition) between consumers who selected a given CATA term and those who did not. Positive values indicate a hedonic lift, whereas negative values indicate a penalty. Only attributes meeting the predefined minimum citation-frequency threshold and showing a statistically significant difference in overall liking are presented.

The penalty/lift analysis further clarified which sensory characteristics were associated with consumer acceptance under blind conditions (Figure 2).

The largest hedonic lifts were observed for “milky” (mean impact = 3.26), “light color” (2.71), and “thick” (2.62), while “creamy” and “sweet” were also associated with significantly higher overall liking. Together, these results indicate that young consumers prefer products with a light, dairy-like appearance and a thick, creamy consistency. In contrast, the strongest penalties were associated with “artificial” (mean impact = −3.08), “bland” (−2.86), and “gray color” (−2.52), while “thin” and “cereal” were also associated with lower liking. Thus, lower young consumer acceptance was linked not only to undesirable flavor characteristics but also to visual and textural cues that were inconsistent with young consumers’ expectations of a yogurt-like product.

Similar sensory drivers of consumer acceptance have been identified in previous studies using different sensory profiling approaches. Based on CATA profiling and consumer liking ratings, Jaeger et al. [34] showed that a smooth and creamy mouthfeel together with vanilla flavor were positively associated with consumer acceptance, whereas sourness, a lumpy appearance, and non-white color contributed to lower liking. Greis et al. [28], using Rate-All-That-Apply (RATA) and Just-About-Right (JAR) methodologies combined with regression analyses, likewise identified creaminess as the main textural driver of liking among plant-based yogurt alternatives, while watery and slimy mouthfeel were negatively associated with consumer acceptance. Gupta et al. [48], integrating CATA with compositional, textural, and consumer data using multiple factor analysis, also demonstrated that dairy and plant-based yogurt alternatives associated with “good texture”, “smooth”, and “creamy” received higher overall liking. Similarly, Brückner-Gühmann et al. [49] showed, for fermented oat-based gels developed as yogurt alternatives, that sweet, moist, soft, and smooth sensations were positively related to overall liking, while penalty analysis identified creaminess as a positive driver of liking. These findings are further supported by Greis et al. [13], who demonstrated that temporal texture perception strongly influenced consumer acceptance. In their study, thickness and creaminess emerged as the main positive drivers of liking, whereas thinness and wateriness were associated with lower consumer acceptance. Collectively, these studies support the present findings, indicating that consumer acceptance of plant-based yogurt alternatives is driven by a combination of dairy-like appearance, adequate thickness, creaminess, and favorable flavor characteristics. Furthermore, the sensory quality of plant-based fermented products is strongly linked to the raw materials used, but as studies by other authors show, it can also be modified through the recipe composition and production process.

### 3.4. Integrated Relationships Between Physicochemical Characteristics, Sensory Perception, and Consumer Acceptance

**Figure 3:**
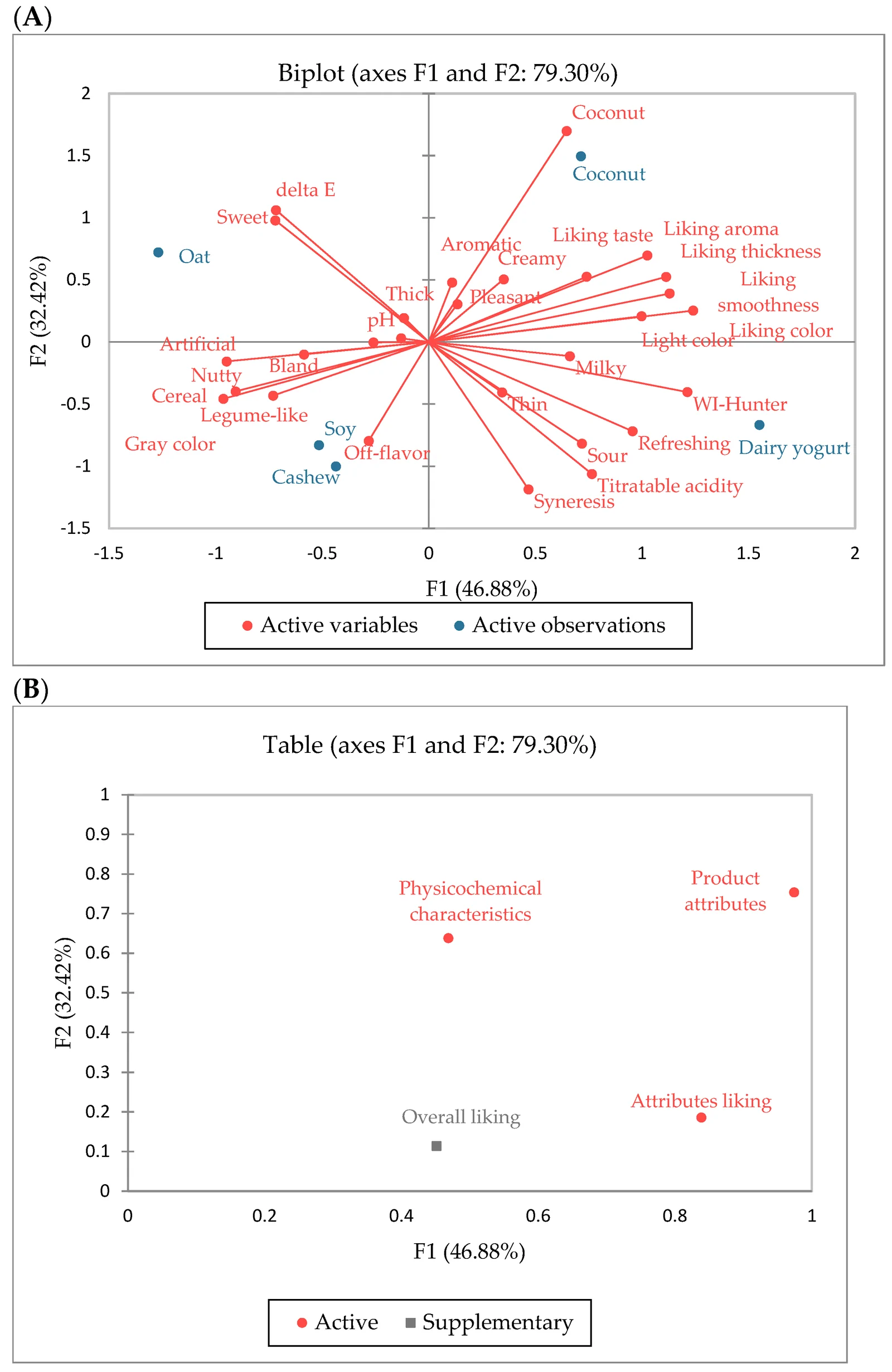
Multiple factor analysis integrating consumer and product data: (**A**) biplot showing the relationships among the evaluated products, sensory and physicochemical variables, and supplementary overall liking; (**B**) representation of the three active data blocks and supplementary overall liking on the first two MFA dimensions.

Multiple factor analysis was performed to integrate three complementary data blocks describing the evaluated products: liking ratings of individual sensory quality attributes, physicochemical characteristics, and CATA-derived sensory profiles. Overall liking was included as a supplementary variable and therefore did not contribute to the construction of the MFA dimensions. The first two dimensions explained 79.30% of the total variability, with F1 and F2 accounting for 46.88% and 32.42%, respectively (Figure 3A).

Dairy yogurt was positioned close to overall liking and to the positive direction of the attribute-liking variables. Young consumers’ frequent description of this product as “sour” was consistent with its highest titratable acidity, while its high Hunter Whiteness Index corresponded to frequent selection of “light color” and high color-liking scores (Table 3, Table 4 and Table 5; Figure 1), together with the “milky” and “refreshing” characteristics. These results indicate that the high acceptance of dairy yogurt was associated with a combination of a light appearance, a familiar dairy-like sensory profile and favorable hedonic evaluations.

The coconut-based alternative showed a different but similarly favorable combination of characteristics. Its low syneresis aligned with a creamy sensory profile and with the highest liking of thickness and smoothness (Table 3 and Table 4; Figure 3A). The product was also described as “coconut” and “aromatic” and received relatively high aroma and taste liking. Despite its large instrumental color difference from dairy yogurt (Δ*E* = 13.16), young consumers frequently described it as having a “light color” and rated it most favorably. These findings indicate that a large instrumental color difference does not necessarily translate into lower visual acceptance when the resulting appearance is perceived positively by young consumers.

The oat-based alternative exhibited the lowest titratable acidity and was strongly associated with “sweet”, whereas “sour” was selected only infrequently (Table 3; Figure 1). It also showed the largest color difference from dairy yogurt (Δ*E* = 13.90) and the lowest Hunter Whiteness Index, which corresponded to frequent selection of “gray color” and the lowest color-liking score among all products (Table 3, Table 4 and Table 5). Although no syneresis was detected, young consumers gave relatively low ratings for thickness and smoothness. The absence of visible phase separation therefore did not necessarily translate into favorable textural perception. At the same time, taste liking was comparable to that of the coconut-based alternative, demonstrating that the lower acceptance of the oat-based product was more closely connected with its appearance and texture than with taste.

The cashew- and soy-based alternatives were positioned opposite to overall liking and most attribute-liking variables. The cashew-based product combined the highest syneresis with less favorable flavor-related characteristics, whereas the soy-based alternative was characterized by the descriptors “legume-like”, “artificial”, “off-flavor”, and “gray color”, which corresponded with low taste liking and overall acceptance (Table 3, Table 4 and Table 5; Figure 3A). For both products, young consumer rejection appeared to be driven primarily by the combined effect of unfavorable sensory characteristics rather than any single physicochemical parameter.

The representation of the data blocks confirmed their complementary contribution to product differentiation (Figure 3B). The strongest agreement was observed between attribute liking and CATA profiles (RV = 0.796), followed by CATA profiles and physicochemical characteristics (RV = 0.679), whereas agreement between physicochemical characteristics and attribute liking was weak (RV = 0.195). These findings suggest that the influence of physicochemical properties on young consumer responses was mediated largely through the sensory perception. Overall, young consumer acceptance appeared to depend more on the integration between quality attributes, sensory perception, and hedonic responses rather than on individual product characteristics considered separately.

Relationships between consumer-perceived sensory characteristics and instrumental product properties have also been examined using multivariate approaches. Ghanbari et al. [50] studied nine formulations of a prebiotic cereal-based dairy dessert and related consumer CATA responses to rheological measurements. The authors found that formulation differences were reflected in both sensory and rheological properties, with both approaches providing similar information regarding product differentiation. Although the instrumental characterization used by Ghanbari et al. [50] included detailed rheological measurements and therefore differed from the physicochemical characteristics assessed in the present study, their findings illustrate the complementary information that sensory and instrumental characterization can provide when examining differences among products. A broader range of consumer and product-related characteristics was investigated by Gupta et al. [48] in commercial dairy and plant-based yogurts, including consumer acceptance, sensory characteristics, product-related attributes, composition, and textural properties. Their MFA showed that overall liking was positively associated with protein content, gel firmness, and the consistency coefficient, whereas fat, sugar, and calorie content showed negative relationships with liking. In the present study, however, the relatively low RV coefficient between sensory attribute liking and physicochemical characteristics (RV = 0.195) indicated limited similarity between the multivariate configurations of these two data blocks, whereas greater similarity was observed between sensory attribute liking and CATA profiles (RV = 0.796).

### 3.5. Effect of Ingredients and Nutritional Information on Consumer Acceptance

Young consumer acceptance was significantly affected by product information, as evidenced by a significant product × condition interaction (F(8.608) = 10.85; *p* < 0.001). Ingredients and nutritional information increased the liking scores for most products, with the largest expectation effects observed for the cashew-based alternative and soy-based alternative (E − B = +2.12 and +1.29, respectively; *p* < 0.001; Table 6).

Smaller but significant increases were found for the coconut-based alternative and dairy yogurt, whereas expected liking of the oat-based alternative did not change significantly (Table 6). In the informed condition, liking remained significantly higher than under blind conditions for dairy yogurt (7.0 vs. 6.4; I-B = +0.65; *p* < 0.05) and the coconut-based alternative (5.8 vs. 5.1; I-B = +0.70; *p* < 0.05). Informed liking did not differ significantly from expected liking for either product, indicating that the positive expectations induced by product information were confirmed during tasting (Table 6).

**Figure 4:**
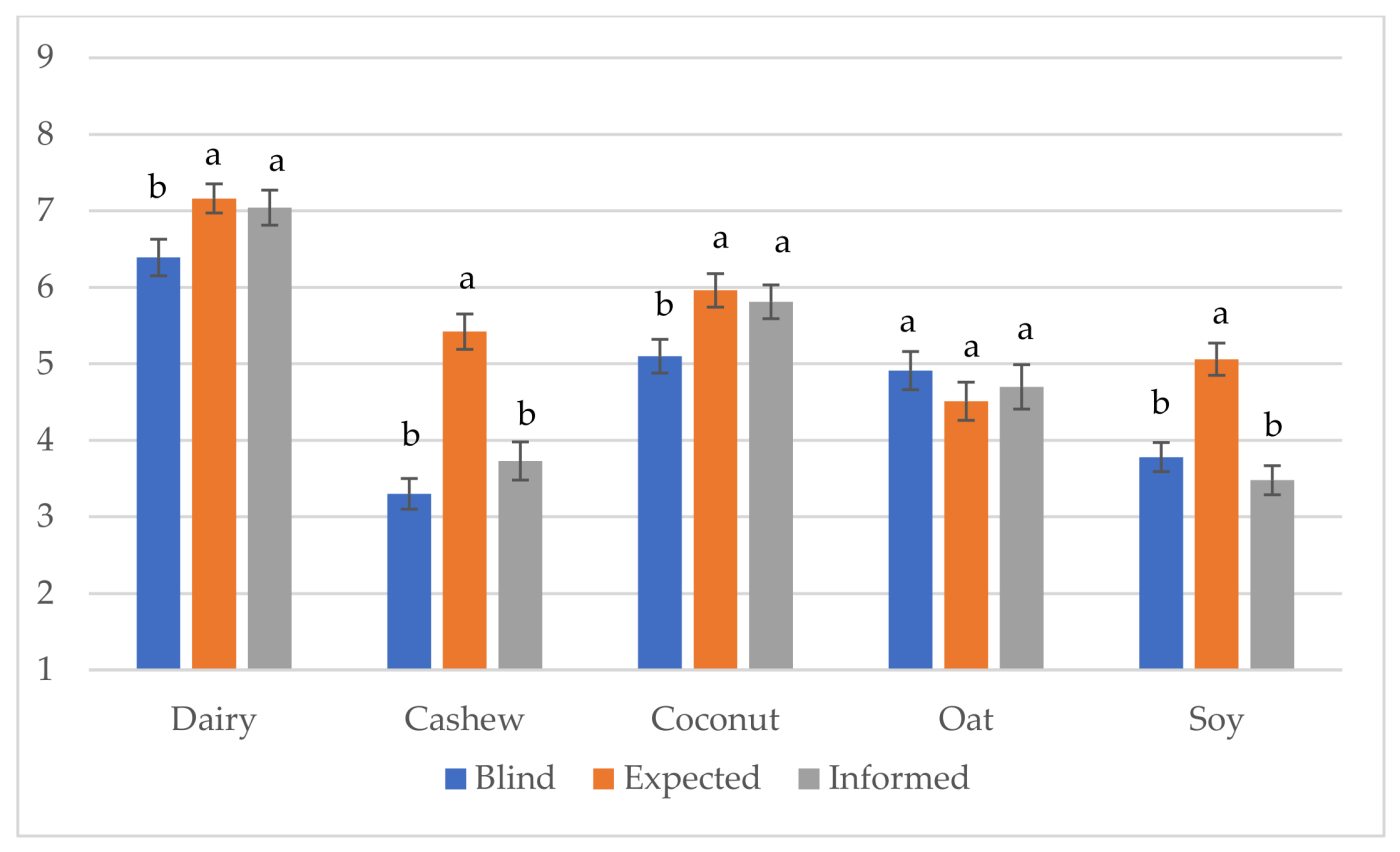
Overall liking of dairy yogurt and plant-based yogurt alternatives under blind, expected, and informed conditions. Values are means ± SEs. For each product, different letters indicate significant differences between evaluation conditions according to Holm-adjusted pairwise comparisons; *p* < 0.05.

In contrast, the positive effect of information was not sustained for the cashew- and soy-based alternatives. Although ingredients and nutritional information generated favorable expectations, these were not confirmed by the sensory experience. Young consumers’ liking of the cashew-based alternative decreased significantly between the expected and informed conditions (5.4 vs. 3.7; I-E = −1.69; *p* < 0.001), returning to a level not significantly different from blind tasting. A similar pattern was observed for the soy-based alternative (Figure 4, Table 6). For these two alternatives, the sensory evaluation resulted in a clear disconfirmation of expectations, limiting the positive influence of product information on acceptance. Liking of the oat-based alternative remained stable across all three conditions (Figure 4, Table 6). Ingredient and nutritional information can shape young consumer expectations, but its impact on final acceptance depends on the extent to which those expectations were confirmed by the sensory experience.

Bayarri et al. [51] similarly demonstrated that the influence of product and nutritional information on the acceptability of yogurts and fermented milk products depended on the extent to which expectations were confirmed by sensory experience and on individual consumer preferences. Consistent with the present findings, Jaeger et al. [18] reported that factual health and environmental information had only a minimal impact on consumer responses to commercial plant-based yogurt alternatives and concluded that improving sensory quality is essential, as product information alone is insufficient to substantially change consumer acceptance when sensory quality is poor.

To better understand individual consumer responses to ingredient and nutritional information, the expectation effects were analyzed according to the approach proposed by Bayarri et al. [51]. Assimilation responses indicate that product evaluations moved toward prior expectations, whereas contrast responses reflect rejection of those expectations following tasting. The distribution of responses differed markedly among products (Table 7).

No-effect or unclear responses were the most frequent for dairy yogurt (45.5%). Assimilation responses predominated for the coconut- and cashew-based alternatives (55.8% and 50.6%, respectively; Table 7), indicating that information about the product ingredients and nutritional value substantially influenced young consumers’ evaluation of these products. At the same time, the cashew-based alternative also elicited a high proportion of contrast responses (20.8%), suggesting that for a group of young consumers, the sensory experience failed to meet the expectations generated by the product information. Responses to the oat-based alternative were more evenly distributed across assimilation (39.0%), contrast (23.4%), and no-effect or unclear responses (37.7%), suggesting the absence of a dominant response pattern. For the soy-based alternative, assimilation and no-effect or unclear responses occurred with similar frequency (42.9% and 41.6%, respectively), suggesting that the influence of information varied considerably across young consumers. The individual-level analysis revealed considerable heterogeneity in young consumer responses, demonstrating that the impact of ingredient and nutritional information depended not only on the product characteristics but also on how individual young consumer integrated this information with sensory experience. Similar heterogeneity was reported by Bayarri et al. [51], who showed that average acceptability scores of yogurts and fermented milk products could mask substantial differences in consumer responses and that a given product could elicit assimilation in one consumer preference subgroup and contrast in another.

### 3.6. Purchase Intention Before and After Tasting

Purchase intention was assessed under two conditions: expected, when consumers based their decision solely on ingredient and nutritional information, and informed, when both product information and sensory experience were available. Young consumers significantly differentiated among the evaluated products under both conditions, as confirmed by the Friedman test for expected purchase intention (χ^2^(4) = 85.92, *p* < 0.001) and informed purchase intention (χ^2^(4) = 133.65, *p* < 0.001).

When young consumers relied only on ingredients and nutritional information, dairy yogurt generated the strongest purchase intention (4.12 ± 0.12), whereas the oat-based alternative generated the weakest response (2.34 ± 0.13) (Table 8).

Among plant-based alternatives, young consumers declared the highest expected purchase intention for the coconut-based alternative (3.13 ± 0.16), followed by the cashew- and soy-based alternatives (2.91 ± 0.12 and 2.68 ± 0.14, respectively). This suggests that before tasting, product information created relatively more favorable purchase expectations for coconut-, cashew-, and soy-based alternatives than for the oat-based product.

The tasting phase may substantially modify young consumers’ purchase intentions because sensory experience allows for the verification of pre-existing expectations. After tasting the products with information available, young consumers maintained the highest purchase intention for dairy yogurt (4.18 ± 0.11). Purchase intention for dairy yogurt did not differ significantly between expected and informed conditions (ΔPI = +0.06, *p* > 0.05), indicating that tasting did not weaken the purchase response generated by product information. A similarly stable response was observed for the coconut-based alternative, for which purchase intention remained almost unchanged after tasting (3.13 ± 0.16 vs. 3.06 ± 0.14; ΔPI = −0.06, *p* > 0.05, Table 8). A similar pattern was observed for the oat-based alternative. Although young consumers initially declared the lowest expected purchase intention for this product, tasting increased their willingness to buy it from 2.34 ± 0.13 to 2.75 ± 0.14 (ΔPI = +0.42, *p* < 0.01, Table 8). This suggests that the sensory experience improved the purchase-related response to the oat-based alternative, despite its relatively weak information-based evaluation. As reported previously, taste is one of the primary drivers of young consumers’ purchase decisions for plant-based beverages, exceeding the importance of health benefits, raw material, nutritional value, and freshness [52]. The positive relationship between overall liking and purchase intention is consistent with Mustapa et al. [53], whose model identified both informed tasting and sensory perceptions as significant factors associated with consumers’ intention to purchase plant-based products. Consistent with these findings, the present results suggest that sensory experience may either reinforce or weaken purchase intention depending on whether the product meets consumers’ expectations. While tasting improved the willingness to purchase the oat-based alternative, it reduced the purchase intention for the cashew- and soy-based alternatives, indicating that sensory characteristics played an important role in shaping young consumer’s purchase intention.

For the cashew-based alternative, purchase intention decreased from 2.91 ± 0.12 under expected conditions to 2.23 ± 0.13 under informed conditions (ΔPI = −0.68, *p* < 0.001). A comparable decrease was observed for the soy-based alternative, for which purchase intention declined from 2.68 ± 0.14 to 2.04 ± 0.11 (ΔPI = −0.64, *p* < 0.001).

Our results show that ingredients and nutritional information alone did not fully predict young consumers’ final purchase intention. Nevertheless, information highlighting specific nutritional benefits may encourage purchase intention. Brückner-Gühmann et al. [49] found that consumers expressed greater willingness to purchase fermented oat-based gels when information about oat protein enrichment was provided than under blind tasting conditions. Similarly, Morris et al. [54] showed that health-related information could modify purchase intention, reporting a 2.6-fold increase for the Frontière white rice flour-based yogurt alternative after a health claim was provided.

The relationship between consumer acceptance and purchase intention was analyzed to determine whether higher overall liking translated into a stronger willingness to buy the evaluated products. Spearman’s rank correlation coefficients were calculated separately for each product under expected and informed conditions (Table 9).

Positive and significant associations between overall liking and purchase intention were observed for all products in both evaluation conditions. Under expected conditions, the correlations ranged from ρ = 0.564 for dairy yogurt to ρ = 0.748 for the soy-based alternative. Under informed conditions, most correlations were also strong, ranging from ρ = 0.687 to ρ = 0.748, with the exception of the soy-based alternative, for which the association was weaker, although still significant (ρ = 0.490). These results suggest that consumers who expected or experienced higher levels of satisfaction generally indicated in the questionnaire that they would be more likely to purchase the products. However, the weaker informed association observed for soy suggests that, for this alternative, purchase intention may have been additionally limited by product-specific barriers other than liking alone.

Linear mixed-effects models were used to examine the association between overall liking and purchase intention. Similar models have previously been applied in consumer studies involving multiple product evaluations per participant, with the consumer included as a random effect to account for within-subject dependence [55].

Linear mixed-effects models confirmed that overall liking was positively associated with purchase intention under both expected and informed conditions (Table 10).

Under expected conditions, each 1-point increase in overall liking was associated with a 0.427-point increase in purchase intention (B = 0.427, 95% CI: 0.383–0.470, *p* < 0.001). Under informed conditions, each 1-point increase in overall liking was associated with a 0.341-point increase in purchase intention (B = 0.341, 95% CI: 0.301–0.380, *p* < 0.001). The product was a significant fixed effect in both models (*p* < 0.001), indicating that purchase intention differed among products even after accounting for overall liking. This suggests that purchase intention was not determined by acceptance alone but was also influenced by product-specific characteristics.

Changes in overall liking were also positively associated with changes in purchase intention. Each 1-point increase in informed-minus-expected overall liking was associated with a 0.213-point increase in purchase intention (B = 0.213, 95% CI: 0.167–0.259, *p* < 0.001). The product was also a significant fixed effect in the change model (*p* < 0.001). Thus, when tasting improved young consumers’ acceptance, purchase intention tended to increase, whereas a decrease in acceptance was accompanied by lower willingness to buy. This pattern was consistent with the product-specific results: tasting reduced purchase intention for the cashew- and soy-based alternatives, increased it for the oat-based alternative, and did not significantly change purchase intention for natural yogurt or the coconut-based alternative (Table 8).

The relationship between product acceptance and purchase intention should be considered in the context of both sensory experience and available product information. Banovic et al. [56] found that providing nutritional information about oat-protein-enriched foods before the first tasting resulted in higher purchase intention than providing it after tasting. Information presented before tasting also increased the contribution of perceived healthiness to purchase intention, whereas taste played a greater role when information followed tasting.

The importance of sensory acceptance for purchase-related responses is also supported by previous studies. Grasso et al. [57] found that among beef, plant-based, and hybrid beef–vegetable burgers, the hybrid burger received the highest overall liking score under blind conditions and elicited higher purchase intention than the plant-based burger under informed conditions. Similarly, Aksu and Seçim [58], investigating a vegan aquafaba-based cookie, reported that higher overall liking was associated with a more favorable purchase-related response. In the context of plant-based yogurt alternatives, Östlund et al. [59] identified taste as the most important criterion for product choice across all dietary groups, while texture, appearance, and price were also considered important. Among flexitarians who were uncertain about or unwilling to consume these products, unpleasant taste was the most frequently reported barrier. Together, these findings are consistent with the importance of sensory acceptance observed in the present study, in which changes in overall liking were positively associated with changes in purchase intention.

Purchase intention should nevertheless be distinguished from actual purchasing behavior. Kytö et al. [60] found that purchase intentions based on both product expectations and perceptions were significant predictors of subsequent purchasing behavior for dairy products. Their findings support the relevance of stated purchase intention as a purchase-related measure, although such declarations should not be regarded as equivalent to actual purchases.

## 4. Conclusions

Among the evaluated products, dairy yogurt received the highest overall liking under blind conditions, confirming the continued importance of conventional yogurt sensory attributes for young consumer acceptance. Among the plant-based alternatives, the coconut-based fermented product was the most accepted and was characterized by a favorable sensory profile. Taste was the sensory quality attribute most strongly associated with overall liking, while the CATA-based analysis showed that “milky”, “light color”, “thick”, “creamy”, and “sweet” were associated with higher liking, whereas “artificial”, “bland”, “gray color”, “thin”, and “cereal” were associated with lower liking. The MFA further demonstrated the value of integrating physicochemical measurements with sensory perception and hedonic evaluation to explain differences among the evaluated products.

Ingredient and nutritional information generally created more favorable expectations, but its influence differed among products. The strongest positive shifts from blind to expected liking were observed for the cashew- and soy-based alternatives. However, tasting did not confirm these expectations, and informed liking returned to levels similar to those observed under blind conditions. In contrast, the higher expectations generated for dairy yogurt and the coconut-based alternative were maintained after tasting, while acceptance of the oat-based alternative remained relatively stable across conditions. At the individual level, assimilation, contrast, and no-effect responses occurred in different proportions, showing that young consumers differed in how they integrated product information with subsequent sensory experience.

Our findings showed that consumer acceptance was a key determinant of purchase intention, while product information and product-specific differences also shaped young consumers’ purchase-related responses. Changes in liking between expected and informed conditions were reflected in corresponding product-specific changes in purchase intention. Our findings showed that consumer acceptance was a key determinant of purchase intention, while product information and product-specific perceptions also contributed to young consumers’ responses. Changes in liking between expected and informed conditions were accompanied by corresponding changes in purchase intention. Therefore, in the case of plant-based yogurt alternatives, improving sensory acceptance appears essential, but not sufficient; product information should also create expectations that can be supported by the subsequent sensory experience. From a practical perspective, product development should focus on achieving an appealing and coherent sensory profile rather than assuming that all plant-based alternatives should necessarily reproduce conventional dairy yogurt characteristics.

## 5. Limitations

Several limitations should be considered when interpreting the findings of this study. Firstly, the consumer sample consisted predominantly of young adults, which may limit the generalizability of these findings to other consumer populations. Secondly, the study included only a few commercially available dairy and plant-based yogurt alternatives. The evaluated products differed not only in their dairy or plant base but also in their overall formulations, including the use of additional ingredients. Consequently, the observed differences relate to the specific products evaluated and cannot be attributed solely to the plant sources or generalized to entire categories of plant-based yogurt alternatives. Future studies should include more diverse consumer populations and wider range of plant-based yogurt alternatives with more closely controlled formulations., Thirdly, purchase intention was assessed under controlled experimental conditions without a real purchase situation. These intentions may therefore not translate into actual purchasing behavior. Future studies could assess consumers’ choices in real or simulated purchasing settings to examine whether the observed effects of label information extend beyond stated purchase intention.

## Figures and Tables

**Table 1:** Description of the selected fermented products.

Base	Ingredients *	Nutritional Declaration *	Images of Samples Used in the Research
Dairy	full milk, lactic acid bacteria	Energy value 305 kJ/73 kcal, total fat 3 g, saturated fat 2 g, total carbohydrates 6.5 g, sugars 6.5 g, fiber 0 g, protein 4.9 g, salt 0.1 g	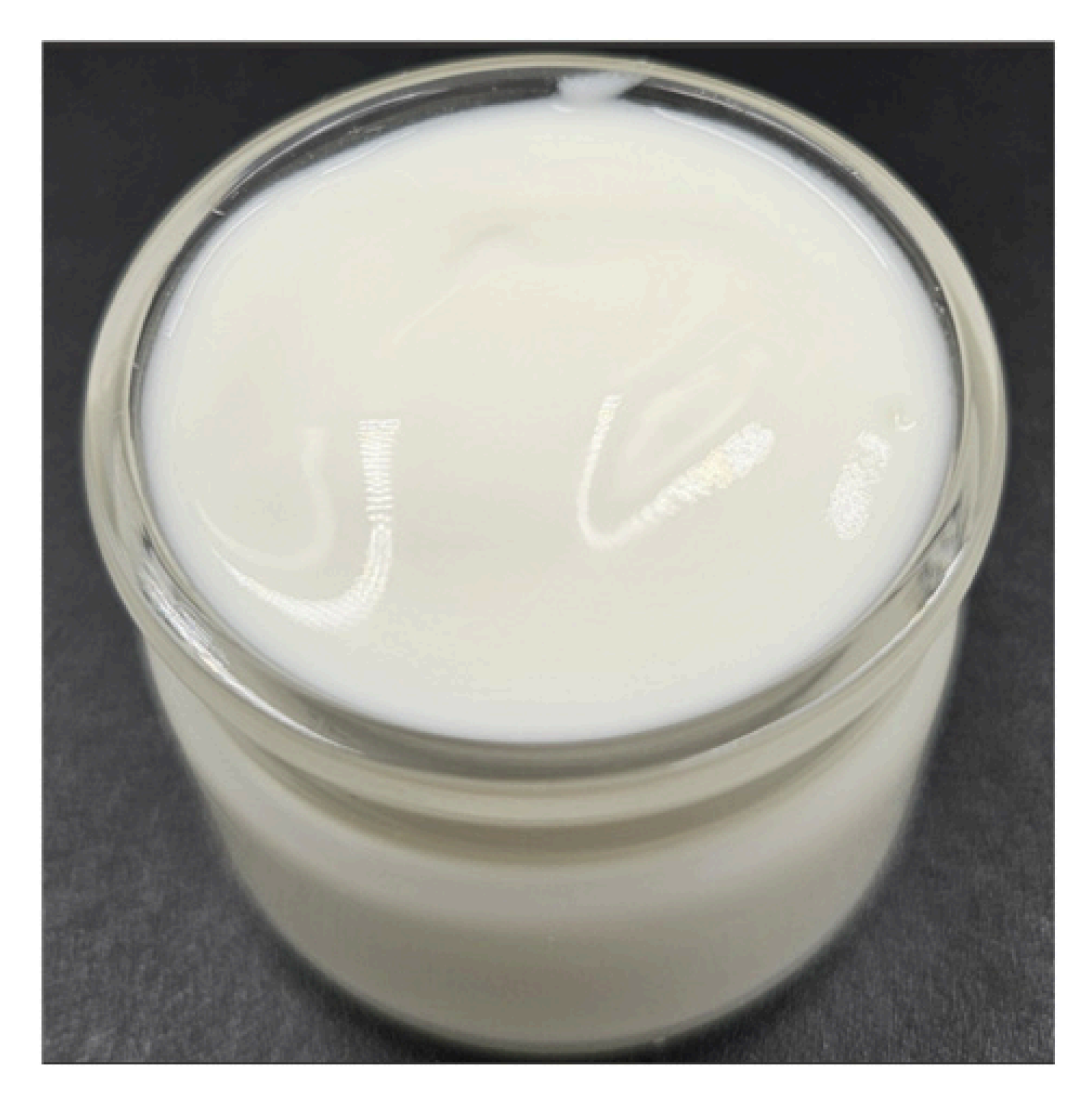
Cashew	cashew drink 92.4% (water, ground cashews), tapioca flour, starch, sunflower protein, inulin, thickening agent (pectin, vegan yogurt cultures)	Energy value 272 kJ/65 kcal, total fat 2.9 g, saturated fat 0.5 g, total carbohydrates 7.4 g, sugars 0.6 g, fiber 1.5 g, protein 1.6 g, salt 0.01 g	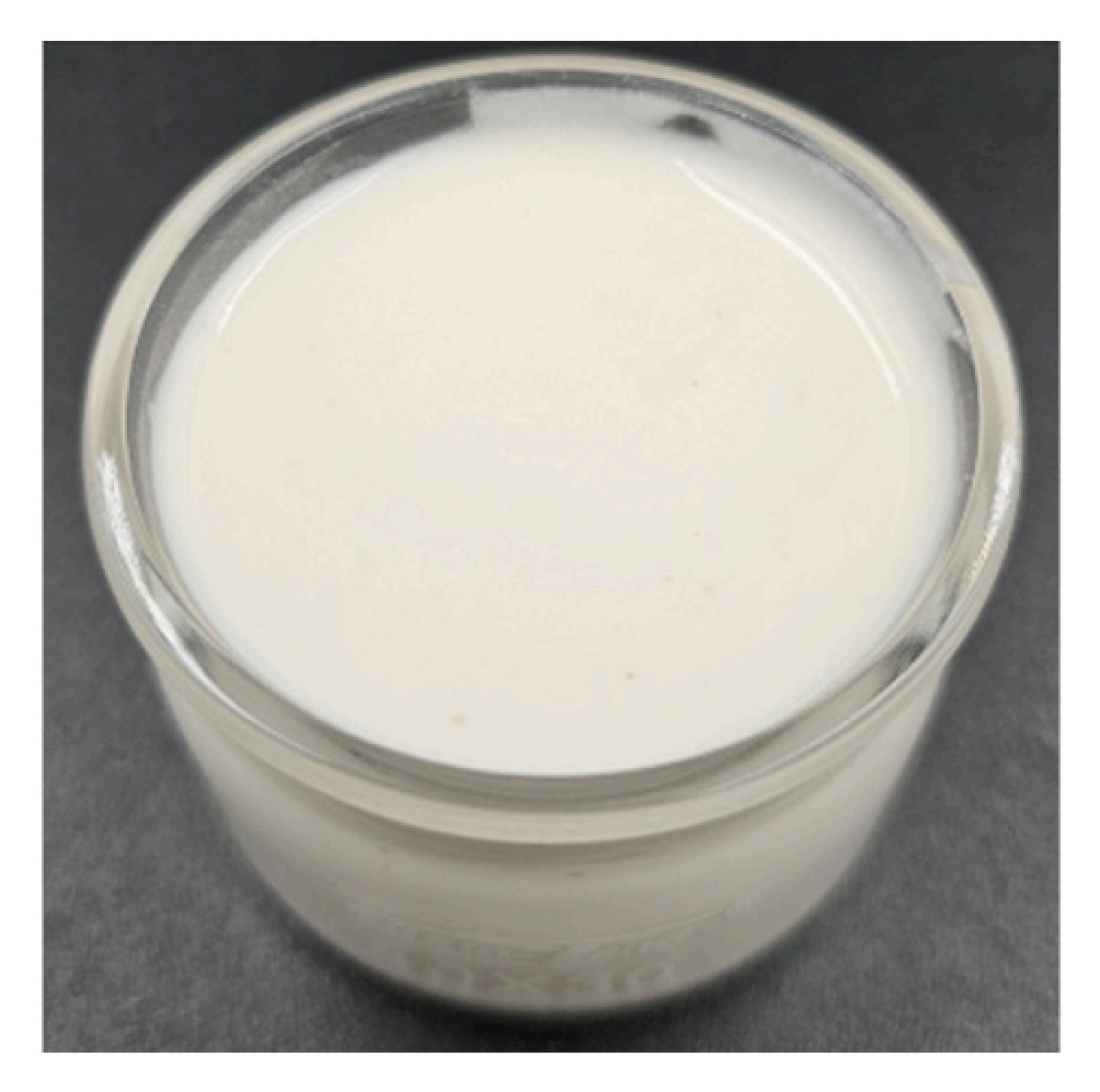
Coconut	organic coconut drink 95.6% (water, organic coconut extract), organic starch, organic carob flour, vegan yogurt cultures (*S. thermophilus, L. delbrueckii* ssp. *bulgaricus*)	Energy value 384 kJ/93 kcal, total fat 7.1 g, saturated fat 6.8 g, total carbohydrates 6.4 g, sugars 0.9 g, fiber <0.5 g, protein 0.7 g, salt 0.03 g	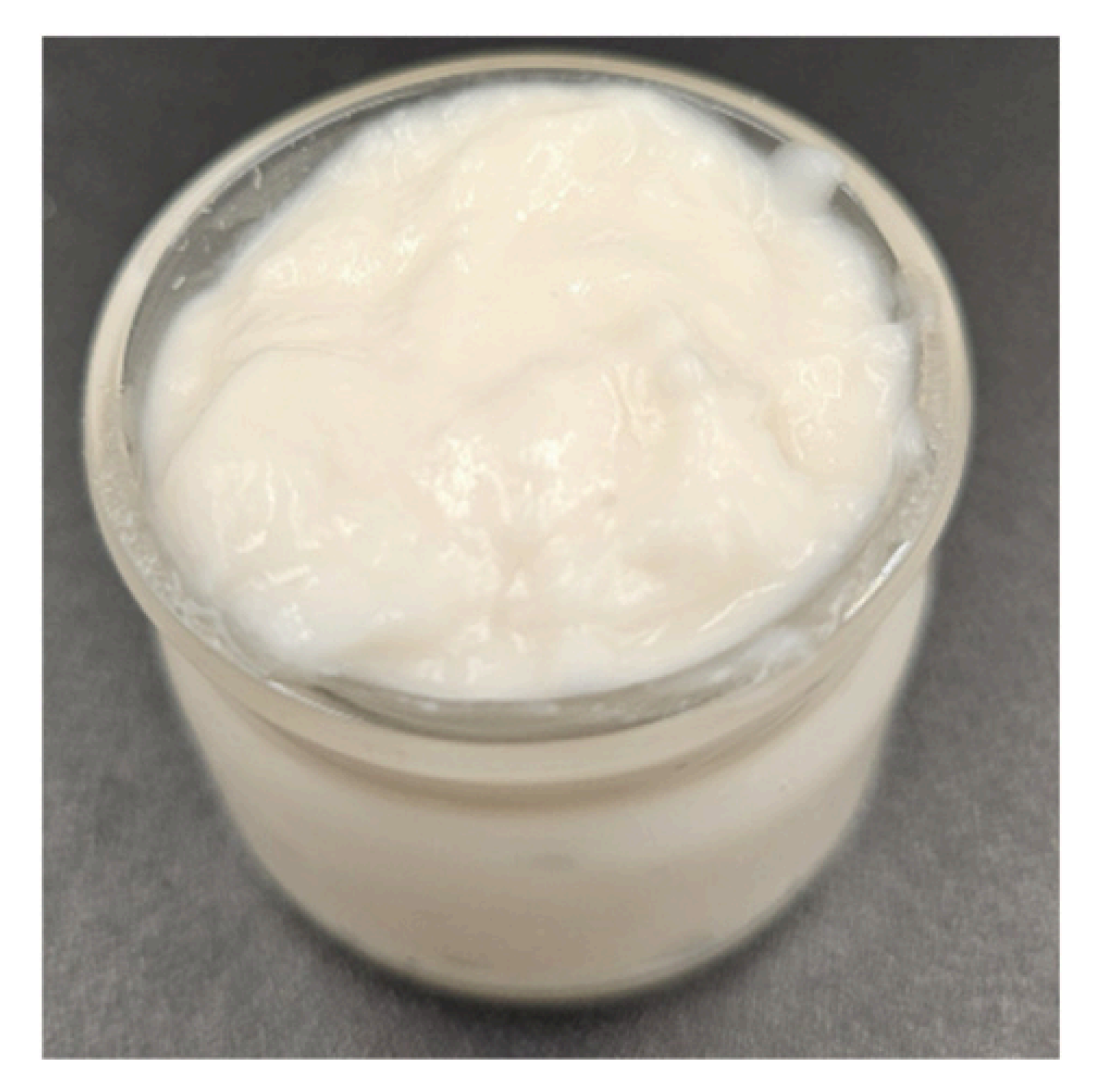
Oat	oat drink (91%) (water, whole oats (11%), yogurt cultures), coconut oil, waxy corn starch, thickening agent: locust bean gum, sea salt (certified organic ingredient)	Energy value 374 kJ/83 kcal, total fat 4.5 g, saturated fat 3.7 g. total carbohydrates 9.8 g, sugars 5.8 g, fiber 0 g, protein 0.7 g, salt 0.1 g	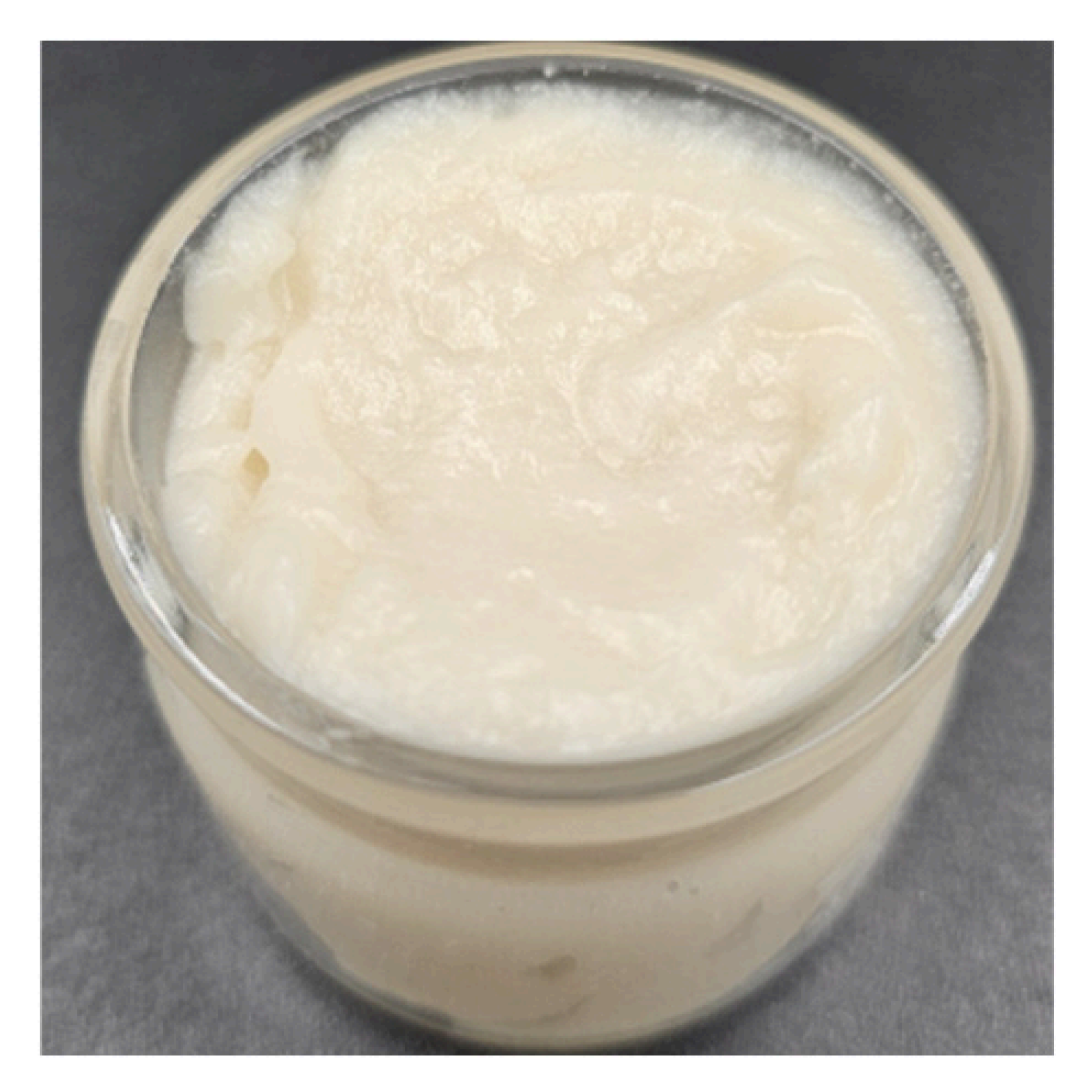
Soy	soy drink (99.8%) (water, hulled soybeans (9.2%)), selected live *Bifidus* and *Acidophilus* cultures	Energy value 190 kJ/46 kcal, total fat 0.4 g, saturated fat 0.4 g, total carbohydrates 0 g, sugars 0 g, fiber 0 g, protein 4.8 g, salt 0.1 g	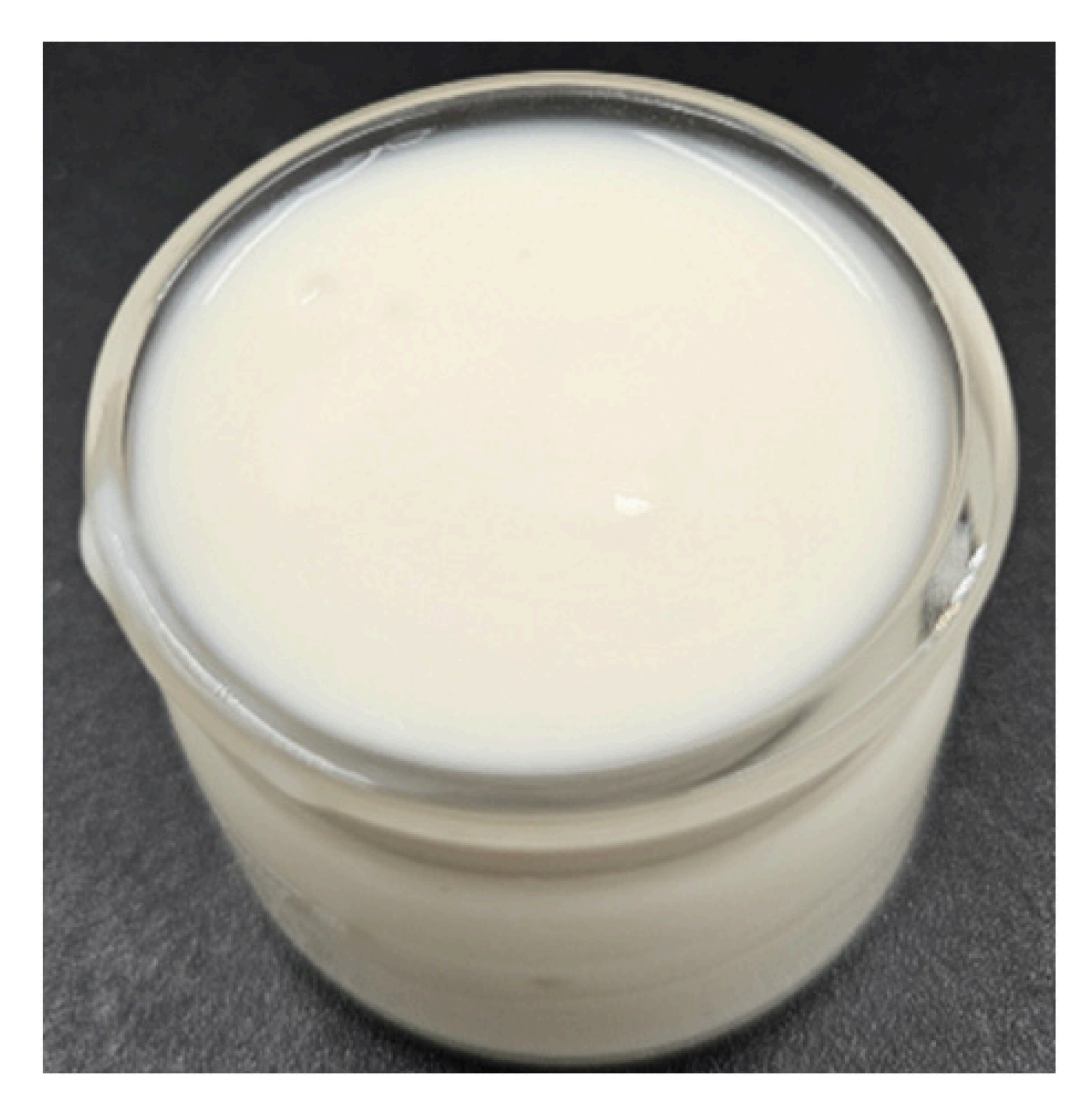

* Producer’s information product label.

**Table 2:** Sociodemographic characteristics of participants.

Feature	Category	Frequency	Percentage
Gender	Female	49	63.5
	Male	28	36.5
Place of residence	Country	27	35
	City below 50,000 citizens	5	6.5
	City between 50,000 and 150,000 citizens	5	6.5
	City between 150,000 and 500,000 citizens	7	9
	City over 500,000 citizens	33	43
Education	Vocational	2	2.5
	Secondary	54	70
	Higher (bachelor/engineer)	19	25
	Higher (master)	2	2.5
Food allergy or lactose intolerant	Yes	77	100.0
	No	0	0.0

**Table 3:** Physicochemical characteristics of selected fermented products.

Base	pH	LA		Color Indices				Syneresis
			L*	a*	b*	Δ*E*	WI	
**Dairy**	4.01 ^c^	1.29 ^a^	74.98 ^a^	−2.98 ^a^	6.65 ^d^	-	55.05 ^a^	25.8 ^b^
**Cashew**	3.97 ^d^	0.89 ^b^	70.29 ^c^	−1.25 ^c^	8.79 ^c^	6.97	43.93 ^c^	27.6 ^a^
**Coconut**	4.15 ^b^	0.29 ^d^	65.68 ^d^	−0.71 ^d^	6.21 ^e^	13.16	47.06 ^b^	3.4 ^d^
**Oat**	4.03 ^c^	0.11 ^e^	61.85 ^e^	−0.69 ^d^	10.56 ^a^	13.90	30.16 ^d^	0.0 ^e^
**Soy**	4.35 ^a^	0.77 ^c^	71.71 ^b^	−2.27 ^b^	9.33 ^b^	5.35	43.71 ^c^	18.4 ^c^

LA—lactic acid %. Values are means of three determinations. Different superscript letters within a column indicate significant differences according to Tukey’s post hoc test (*p* < 0.05). Δ*E* values were calculated from the mean L*, a*, and b* coordinates relative to dairy yogurt and were interpreted as descriptive.

**Table 4:** Overall liking and liking of quality attributes of dairy and plant-based yogurt alternatives under blind conditions.

Base	Overall Liking	Color	Thickness	Smoothness	Aroma	Taste
**Dairy**	6.39 ± 0.24 ^a^	7.34 ± 0.17 ^a^	6.73 ± 0.19 ^b^	6.84 ± 0.19 ^a^	6.00 ± 0.19 ^a^	5.96 ± 0.19 ^a^
**Cashew**	3.30 ± 0.20 ^c^	4.55 ± 0.20 ^b^	5.66 ± 0.19 ^c^	6.04 ± 0.18 ^b^	3.97 ± 0.19 ^c^	2.60 ± 0.19 ^d^
**Coconut**	5.10 ± 0.22 ^b^	7.60 ± 0.17 ^a^	7.44 ± 0.17 ^a^	7.40 ± 0.19 ^a^	5.97 ± 0.18 ^a^	4.78 ± 0.19 ^b^
**Oat**	4.91 ± 0.25 ^b^	3.01 ± 0.20 ^c^	4.73 ± 0.20 ^d^	4.81 ± 0.19 ^c^	4.82 ± 0.20 ^b^	4.81 ± 0.19 ^b^
**Soy**	3.78 ± 0.19 ^c^	4.83 ± 0.19 ^b^	4.71 ± 0.19 ^d^	5.03 ± 0.19 ^c^	4.56 ± 0.20 ^bc^	3.64 ± 0.18 ^c^

Values are means ± SEs (standard errors). Different letters within a column indicate significant differences between products according to Holm-adjusted pairwise comparisons (*p* < 0.05).

**Table 5:** Frequency (%) of the terms used by consumers to describe dairy yogurt and plant-based yogurt alternatives (CATA analysis).

Attribute	Dairy	Cashew	Coconut	Oat	Soy
Sour	92.2	46.8	10.4	5.2	36.4
Sweet	2.6	5.2	41.6	81.8	10.4
Bland	6.5	54.5	51.9	28.6	48.1
Refreshing	27.3	7.8	2.6	2.6	6.5
Artificial	5.2	33.8	19.5	37.7	35.1
Pleasant	27.3	2.6	20.8	31.2	13.0
Milky	49.4	15.6	22.1	18.2	9.1
Cereal	1.3	35.1	2.6	37.7	35.1
Coconut	0.0	1.3	75.3	6.5	3.9
Creamy	29.9	22.1	63.6	26.0	14.3
Aromatic	14.3	3.9	13.0	19.5	1.3
Nutty	1.3	14.3	2.6	27.3	19.5
Legume-like	0.0	14.3	3.9	10.4	16.9
Off-flavor	10.4	29.9	5.2	6.5	32.5
Light color	90.9	16.9	85.7	7.8	24.7
Gray color	1.3	71.4	0.0	68.8	55.8
Thick	42.9	45.5	45.5	71.4	15.6
Thin	39.0	23.4	29.9	3.9	53.2

**Table 6:** Changes in overall liking of dairy yogurt and plant-based yogurt alternatives across blind, expected, and informed conditions.

Base	E − B	I − B	I − E	Interpretation
Dairy	+0.77 **	+0.65 *	−0.12 ns	Expectations sustained
Cashew	+2.12 ***	+0.43 ns	−1.69 ***	Expectations not confirmed
Coconut	+0.75 *	+0.70 *	−0.05 ns	Expectations sustained
Oat	−0.40 ns	−0.21 ns	+0.19 ns	Stable response
Soy	+1.29 ***	−0.30 ns	−1.58 ***	Expectations not confirmed

E − B = Expected − Blind; I − B = Informed − Blind; I − E = Informed − Expected. Significance levels were determined using Holm-adjusted pairwise comparisons; ns, not significant; * *p* < 0.05; ** *p* < 0.01; *** *p* < 0.001.

**Table 7:** Proportion of consumers showing assimilation, contrast, and unclear or no effect of expectations generated by ingredient and nutritional information.

Base	Effect	Consumers [n]	Consumers [%]
Dairy yogurt	Assimilation	33	42.9
	Contrast	9	11.7
	No effect or unclear	35	45.5
Cashew	Assimilation	39	50.6
	Contrast	16	20.8
	No effect or unclear	22	28.6
Coconut	Assimilation	43	55.8
	Contrast	5	6.5
	No effect or unclear	29	37.7
Oat	Assimilation	30	39.0
	Contrast	18	23.4
	No effect or unclear	29	37.7
Soy	Assimilation	33	42.9
	Contrast	12	15.6
	No effect or unclear	32	41.6

Values represent the number and percentage of consumers classified as showing assimilation, contrast, or no effect/unclear response according to the individual-level relationship between expected-minus-blind (E − B) and informed-minus-blind (I − B) overall liking scores.

**Table 8:** Purchase intention of dairy yogurt and plant-based yogurt alternatives under expected and informed conditions.

Base	Expected PI	Informed PI	ΔPI
Dairy	4.12 ± 0.12	4.18 ± 0.11	+0.06 ns
Cashew	2.91 ± 0.12	2.23 ± 0.13	−0.68 ***
Coconut	3.13 ± 0.16	3.06 ± 0.14	−0.06 ns
Oat	2.34 ± 0.13	2.75 ± 0.14	+0.42 **
Soy	2.68 ± 0.14	2.04 ± 0.11	−0.64 ***

Values are means ± SEs. PI, purchase intention; ΔPI = Informed PI—Expected PI. Differences between expected and informed purchase intention were analyzed using Wilcoxon signed-rank test; ns, not significant; ** *p* < 0.01; *** *p* < 0.001.

**Table 9:** Spearman’s correlations between overall liking and purchase intention under expected and informed conditions.

Base	Expected Liking vs. Expected PI	Informed Liking vs. Informed PI
Dairy	ρ = 0.564 ***	ρ = 0.701 ***
Cashew	ρ = 0.631 ***	ρ = 0.691 ***
Coconut	ρ = 0.697 ***	ρ = 0.687 ***
Oat	ρ = 0.721 ***	ρ = 0.748 ***
Soy	ρ = 0.748 ***	ρ = 0.490 ***

PI, purchase intention; *** *p* < 0.001.

**Table 10:** Linear mixed models examining the association between overall liking and purchase intention.

Model	Predictor	B	SE	df	t	95% CI	*p*
Expected PI	Overall liking	0.427	0.022	370	19.327	0.383–0.470	<0.001
Informed PI	Overall liking	0.341	0.020	372	16.801	0.301–0.380	<0.001
ΔPI	Δ Overall liking	0.213	0.023	367	9.109	0.167–0.259	<0.001

PI, Purchase intention; ΔPI and Δ liking were calculated as informed-minus-expected scores; B, unstandardized fixed-effect coefficient; SE, standard error; df, degree of freedom estimated using Satterthwaite’s approximation; t, t-statistic; CI, confidence interval. The product was included as a fixed effect in all models.

## Data Availability

The original contributions presented in this study are included in the article. Further inquiries can be directed to the corresponding author.

## Abbreviations

The following abbreviations are used in this manuscript:

CATA	Check-All-That-Apply (CATA)
LA	Lactic acid
CIELab	Commission Internationale de l’Éclairage L*a*b* color space
WI	Hunter Whiteness Index
REML	Restricted maximum likelihood
CA	Correspondence analysis
RATA	Rate-All-That-Apply
JAR	Just-About-Right
SE	Standard error
